# Self-diagnosis of seasonal influenza in a rural primary care setting in Japan: A cross sectional observational study

**DOI:** 10.1371/journal.pone.0197163

**Published:** 2018-05-10

**Authors:** Hiroki Maita, Tadashi Kobayashi, Hiroshi Osawa, Hiroyuki Kato

**Affiliations:** 1 Development of Community Healthcare, Hirosaki University Graduate School of Medicine, Hirosaki-shi, Aomori, Japan; 2 General Medicine, Hirosaki University Graduate School of Medicine, Hirosaki-shi, Aomori, Japan; 3 Department of General Medicine, Hirosaki University School of Medicine & Hospital, Hirosaki-shi, Aomori, Japan; The University of Hong Kong, CHINA

## Abstract

**Objective:**

To elucidate the accuracy and optimal cut-off point of self-diagnosis and clinical symptoms of seasonal influenza compared with rapid influenza diagnostic tests as the reference standard, we conducted a cross sectional observational study at a rural clinic in Japan.

**Methods:**

Data during three influenza seasons (December 2013 to April 2016) were retrospectively collected from the medical records and pre-examination sheets of 111 patients aged >11 years (mean age 48.1 years, men 53.2%) who were suspected of influenza infection and underwent rapid influenza diagnostic testing. Patients’ characteristics (age, sex, and past medical history of influenza infection), clinical signs (axillary temperature, pulse rate, cough, joint and muscle pain, and history of fever [acute or sudden, gradual, and absence of fever]), duration from the onset of symptoms, severity of feeling sick compared with a common cold (severe, similar, and mild), self-reported likelihood of influenza (%), and results of rapid influenza diagnostic tests.

**Results:**

At the optimal cut-off point (30%) for estimation of self-diagnosis of seasonal influenza, the positive likelihood ratio (LR+) was 1.46 (95% confidence interval 1.07 to 2.00) and negative likelihood ratio (LR–) was 0.57 (0.35 to 0.93). At a 10% cut-off point, LR–was 0.33 (0.12 to 0.96). At an 80% cut-off point, LR+ was 2.75 (0.75 to 10.07). As for clinical signs, the combination of acute or sudden onset fever and cough had LR+ of 3.27 (1.68 to 6.35). Absence of cough showed LR–of 0.15 (0.04 to 0.61).

**Conclusions:**

Self-diagnosis of influenza using the optimal cut-off point (30%) was not found useful for ruling in or ruling out an influenza diagnosis. However, it could be useful when patients self-report extremely high (80%) or low (10%) probability of having influenza. Clinically useful signs were the combination of history of fever and cough, and absence of cough.

## Introduction

Seasonal influenza is a common outpatient problem during the winter in Japan [1]. More than 20 million patients visit Japanese medical institutions annually with influenza, and 0.85/1000 patients are hospitalized [2]. In the U.S., 0.88/1000 influenza-associated hospitalizations occur annually [3]. Seasonal influenza has a great socioeconomic impact owing to its high infectiveness and ability to spread rapidly and extensively. In the U.S., the total economic burden of influenza epidemics in all age groups has been reported to be US$ 87.1 billion [4]. In Japan, the diagnosis of influenza is important in school health policies because schoolchildren with influenza are prohibited from attending school until they can no longer transmit infection to others, according to the School Health and Safety Act. School district closures have been reported to reduce the rate of acute respiratory illness in a community by 45% and decrease emergency department visits owing to influenza [5]. Therefore, early identification and corresponding measures to prevent the spread of influenza are important from a societal perspective.

In Japan, influenza is generally diagnosed using a rapid influenza diagnostic test (RIDT), although it is often clinically diagnosed during influenza epidemics [6]. If clinicians need to distinguish influenza from other diseases, additional laboratory tests are available, such as immunofluorescence assays and reverse transcriptase polymerase chain reaction [7]. RIDTs are reported to have a relatively low sensitivity (62.3%, 95% confidence interval 57.9 to 66.6) [8]. Moreover, the sensitivity of these tests is especially lower when used within 12 hours of the onset of patient symptoms [9]. Despite easier access to medical settings in Japan compared with many other countries, it is difficult to rule out influenza by the use of RIDTs.

Although self-diagnosis of influenza is important to control and manage the spread of influenza, the accuracy of qualitative self-diagnosis has been reported to be poor [10, 11]. For example, the accuracy of qualitative self-diagnosis, which used categorical variables (yes, possibly, no, and don’t know), during the 2009 influenza pandemic in New Zealand had a reported sensitivity of 45.7% (33.0 to 58.3) and specificity of 58.1% (51.0 to 65.3) [10]. However, no published studies have evaluated the quantitative self-diagnosis of influenza, which used continuous variables (%). Therefore, we aimed to elucidate the accuracy and optimal cut-off point of self-diagnosis and clinical symptoms of seasonal influenza compared with the RIDT.

## Materials and methods

We performed a cross sectional observational study to elucidate the clinical effectiveness of self-diagnosis of seasonal influenza at a rural clinic, Towadako Clinic in Towada-shi, Aomori, Japan.

### Data collection

Using the medical records and structured pre-examination checklists (original Japanese version [S1 Fig] and English translated version [S2 Fig]), we retrospectively extracted the data obtained during 3 influenza seasons from December 2013 to March 2016. Participants who were included in the study met all the following criteria: 1) aged 12 years and older, 2) underwent RIDT, and 3) completed the pre-examination checklist by themselves (not a parent or caregiver). Data extracted from the medical records included baseline characteristics (age and sex), clinical signs (axillary temperature obtained at the clinic and pulse rate), and results of RIDT (QuickNavi-Flu, Denka Seiken Co., Ltd., Japan). Information from the checklists included baseline characteristics (past medical history of influenza infection), clinical signs (cough, joint and muscle pain, history of fever [acute or sudden, gradual, and absence of fever], duration from symptom onset to influenza testing, severity of feeling sick compared with a common cold [severe, similar, and mild]), and self-diagnosis presented as percentage (%). The checklist was handed over by a clinic nurse to every patient with any influenza-like symptoms, such as fever, cough, and/or sore throat, at the time of visit.

### Statistical analysis

The receiver operating characteristic (ROC) curve was performed to estimate the optimal cut-off point. It was determined using the Youden index, which was calculated as maximum of sensitivity + specificity—1. Sensitivity, specificity, and the likelihood ratio of self-diagnosis of influenza were determined using multiple cut-off points [12]. To calculate sensitivity, specificity, and the likelihood ratio of clinical symptoms, 2 × 2 tables were analyzed using the RIDT results. All statistical analyses were performed with EZR version 1.32 (Saitama Medical Center, Jichi Medical University, Saitama, Japan) [13], which is a modified version of R Commander that is designed to add statistical functions frequently used in biostatistics.

### Ethics statement

Full ethical approval was granted by the Medical Ethics Committee of Hirosaki University (number of approval 2016–1078). All data were fully anonymized at the time of data collection, and the committee did not require informed consent. Participation of patients was obtained through an opt-out methodology.

## Results

**Fig 1:**
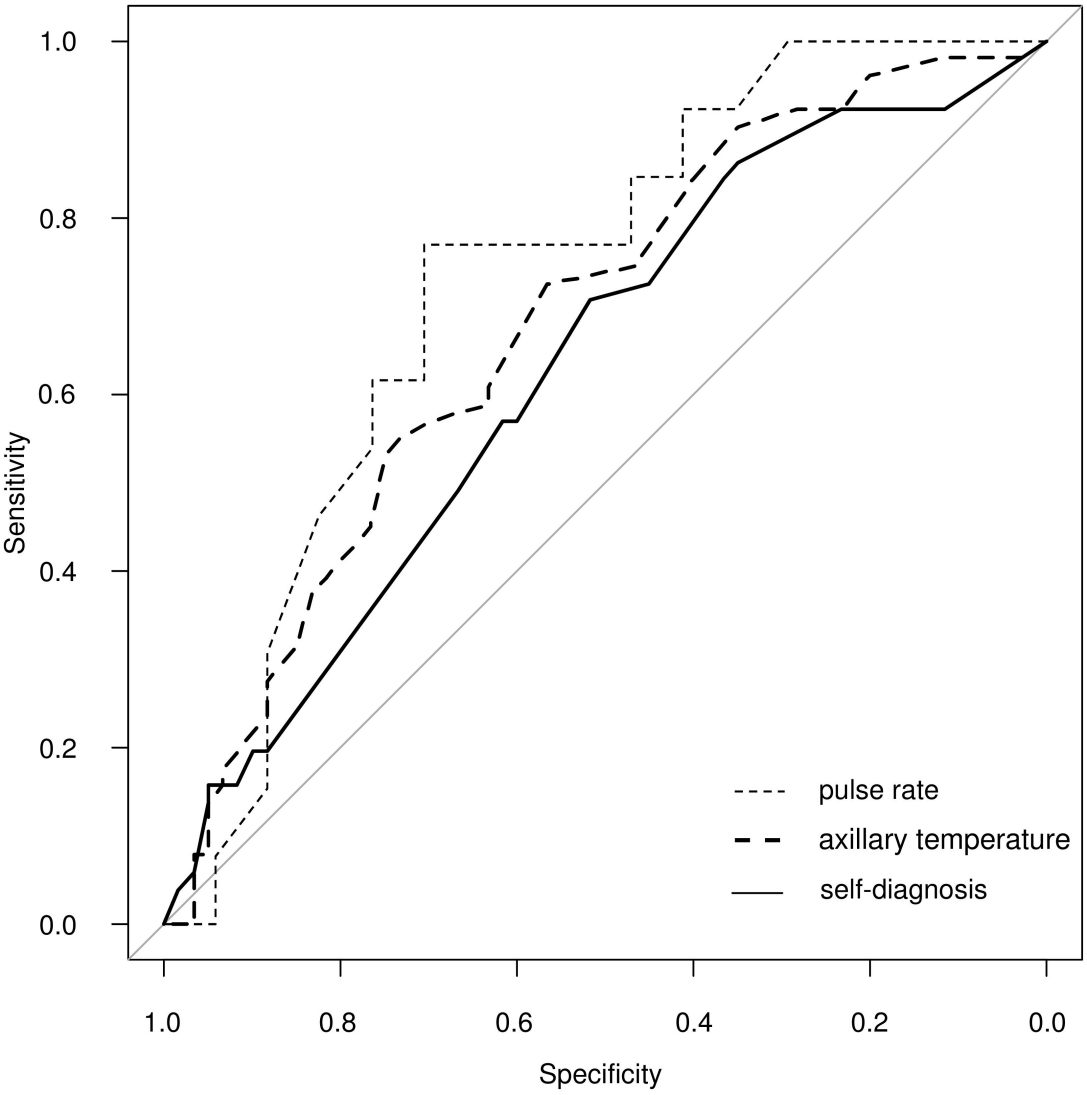
Receiver operating characteristic curve of self-diagnosis, axillary temperature, and pulse rate.

In our study, data for a total 111 patients were analyzed (Table 1). We first estimated the accuracy of self-diagnosis of influenza. The area under the curve (AUC) of self-diagnosis (%) was 0.63 (0.53 to 0.73) (Fig 1). The optimal cut-off point was 30%, at which the sensitivity was 70.6% (56.2 to 82.5), the specificity was 51.7% (38.4 to 64.8), the positive likelihood ratio (LR+) was 1.46 (1.07 to 2.00), and the negative likelihood ratio (LR–) was 0.57 (0.35 to 0.93). At a 10% cut-off point, the LR–was 0.33 (0.12 to 0.96); at an 80% cut-off point, the LR+ was 2.75 (0.75 to 10.07) (Table 2). In the subgroup who had been previously infected with influenza (n = 36), the accuracy of self-diagnosis at a 30% cut-off point was estimated to have a sensitivity of 68.8% (41.3 to 89.0), specificity of 45.0% (23.1 to 68.5), LR+ of 1.25 (0.75 to 2.09), and LR–of 0.69 (0.29 to 1.66). No significant differences were observed between the age groups in terms of AUC: 0.65 (0.32 to 0.98) for 12–17 years; 0.65 (0.51 to 0.78) for 18–64 years; 0.55 (0.24 to 0.87) for 65 years and older.

**Table 1:** Patient characteristics (n = 111).

Mean age, y (range, SD)	48.1(12 to 93, 21.0)
12 to 17, n (%)	15 (13.5)
18 to 64, n (%)	69 (62.2)
>65, n (%)	27 (24.3)
Sex, n (%)	
male	59 (53.2)
female	52 (46.8)
Past history of influenza, n (%)	36 (48.0)
Mean axillary temperature on arrival, °C (SD)	36.9 (0.80)
Pulse rate, beats/min (SD)	90.5 (17.9)
Cough, n (%)	93 (83.8)
Joint and muscle pain, n (%)	68 (61.2)
History of fever, n (%)	
acute or sudden	43 (38.7)
gradual	42 (37.8)
no fever	26 (23.4)
Acute or sudden onset fever + cough	34 (30.6)
Duration from symptom onset to rapid influenza test, h (SD)	52.3 (46.4)
<12 h, n (%)	5.0 (4.5)
≥12 h, n (%)	106.0 (95.5)
Severity of feeling sick compared with common cold, n (%)	
severe	58 (52.3)
similar	39 (35.1)
mild	14 (12.6)
Positive for influenza test, n (%)	51 (45.9)
Significant clinical event requiring hospitalization, n (%)	0 (0.0)

Note: Items not described in the medical record were counted as "none". Abbreviation: SD, standard deviation.

**Table 2:** Receiver operating characteristic curve analysis of influenza self-diagnosis.

Cut-off	Sn,% (95%CI)	Sp,% (95%CI)	LR+ (95%CI)	LR–(95%CI)
0.1	92.2 (81.1 to 97.8)	23.3 (13.4 to 36.0)	1.20 (1.02 to 1.41)	0.33 (0.12 to 0.96)
0.2	84.3 (71.4 to 93.0)	36.7 (24.6 to 50.1)	1.33 (1.06 to 1.67)	0.43 (0.21 to 0.88)
0.3	70.6 (56.2 to 82.5)	51.7 (38.4 to 64.8)	1.46 (1.07 to 2.00)	0.57 (0.35 to 0.93)
0.4	56.9 (42.2 to 70.7)	61.7 (48.2 to 73.9)	1.48 (0.99 to 2.21)	0.70 (0.48 to 1.02)
0.5	49.0 (34.8 to 63.4)	66.7 (53.3 to 78.3)	1.47 (0.93 to 2.32)	0.77 (0.55 to 1.06)
0.6	19.6 (9.8 to 33.1)	90.0 (79.5 to 96.2)	1.96 (0.77 to 5.02)	0.89 (0.76 to 1.05)
0.7	15.7 (7.0 to 29.0)	91.7 (81.6 to 97.2)	1.88 (0.66 to 5.40)	0.92 (0.80 to 1.06)
0.8	13.7 (5.7 to 26.3)	95.0 (86.1 to 99.0)	2.75 (0.75 to 10.07)	0.91 (0.80 to 1.03)

Abbreviations: Sn, sensitivity; Sp, specificity; LR+, positive likelihood ratio; LR–, negative likelihood ratio; CI, confidence interval.

Secondly, in the subgroup with sudden onset fever (n = 43), the sensitivity was 77.8% (57.7 to 91.4), specificity was 50.0% (24.7 to 75.3), the LR+ was 1.56 (0.92 to 2.64), and LR–was 0.44 (0.19 to 1.05) at the optimal cut-off point of 30%. Furthermore, in another subgroup who reported their level of feeling sick to be severe compared with a common cold (n = 58), the sensitivity was 76.5% (58.8 to 89.3), specificity was 70.8% (48.9 to 87.4), the LR+ was 2.62 (1.37 to 5.03), and LR–was 0.33 (0.17 to 0.64) at the optimal cut-off point of 30%.

Thirdly, we validated the accuracy of axillary temperature and pulse rate. The AUC of axillary temperature was 0.68 (0.58 to 0.78) (Fig 1)**.** The difference between self-diagnosis and axillary temperature was not significant (Z = –0.74, P = 0.461). The AUC of pulse rate was 0.73 (0.55 to 0.92) (Fig 1), and the difference between self-diagnosis and pulse rate was not significant (Z = –1.58, P = 0.11). The optimal cut-off point of pulse rate was at 90 beats/min, at which the sensitivity was 76.9% (46.2 to 95.0), the specificity was 70.6% (44.0 to 89.7), the LR+ was 2.62 (1.18 to 5.79), and the LR–was 0.33 (0.12 to 0.92). DeLong’s test under a Bonferroni correction for comparing correlated AUC was performed twice, which did not change the results.

Finally, we examined the accuracy of other clinical signs (Table 3). The combination of acute or sudden onset fever and cough showed an LR+ of 3.27 (1.68 to 6.35). The absence of cough had an LR–of 0.15 (0.04 to 0.61).

**Table 3:** Accuracy of the clinical symptoms of influenza.

	Sn,% (95%CI)	Sp,% (95%CI)	LR+ (95%CI)	LR–(95%CI)
Cough	96.1 (86.5 to 99.5)	26.7 (16.1 to 39.7)	1.31 (1.11 to 1.54)	0.15 (0.04 to 0.61)
Joint and muscle pain	47.6 (34.9 to 60.6)	48.8 (33.3 to 64.5)	0.93 (0.63 to 1.38)	1.07 (0.73 to 1.58)
History of fever	84.3 (71.4 to 93.0)	30.0 (18.8 to 43.2)	1.20 (0.98 to 1.48)	0.52 (0.25 to 1.10)
Acute or sudden onset fever	52.9 (38.5 to 67.1)	73.3 (60.3 to 83.9)	1.99 (1.21 to 3.25)	0.64 (0.46 to 0.89)
plus cough	49.0 (34.8 to 63.4)	85.0 (73.4 to 92.9)	3.27 (1.68 to 6.35)	0.60 (0.45 to 0.80)
Severity of patient feeling sick^¶^				
severe	66.7 (52.1 to 79.2)	60.0 (46.5 to 72.4)	1.67 (1.16 to 2.40)	0.56 (0.36 to 0.86)
similar	23.5 (12.8 to 37.5)	55.0 (41.6 to 67.9)	0.52 (0.30 to 0.92)	1.39 (1.06 to 1.83)
mild	9.8 (3.3 to 21.4)	85.0 (73.4 to 92.9)	0.65 (0.23 to 1.83)	1.06 (0.92 to 1.22)

Abbreviations: Sn, sensitivity; Sp, specificity; LR+, positive likelihood ratio; LR–, negative likelihood ratio, CI, confidence interval.

^¶^ As compared with a common cold.

## Discussion

Our study suggests that self-diagnosis of seasonal influenza would be useful when the patient reports extremely high (80%) or low (10%) probability of being infected with influenza, although the ability to rule in or rule out an influenza diagnosis at the optimal cut-off point (30%) was insufficient. In addition, the LR+ for acute or sudden onset fever adding to cough was relatively high, and the LR–for absence of cough was relatively low.

### Diagnosis of influenza

Reverse transcription polymerase chain reaction is the gold standard for diagnosing influenza infection. However, this test is usually difficult to carry out, especially in rural clinics. Therefore, a diagnosis of influenza is commonly made on the basis of RIDT results, the sensitivity and specificity of which have been reported to be 62.3% (57.9 to 66.6) and 98.2% (97.5 to 98.7), respectively [8]. This test has a relatively low sensitivity, especially when used within 12 hours from the onset of patient symptoms [9].

In Japan, despite a negative RIDT result, patients who are strongly suspected to be infected with influenza are usually re-examined after a sufficient time has passed from the onset of their symptoms. In our study, the average duration from symptom onset to performing the RIDT was 52.3 hours. Nearly all patients (about 95%) were examined more than 12 hours from the time of onset. Three patients were later re-examined and none had positive test results. However, our study data were retrospectively extracted from the medical charts and pre-examination sheets of a single medical institution. In the Japanese system of free-access for patients to medical institutions, patient follow-up tends to be insufficient. In our study, definitive diagnostic information at follow-up might be absent, especially when influenza diagnostic tests were negative upon visiting the study clinic.

### Self-diagnosis and clinical symptoms

We found that the AUC of self-diagnosis was 0.63, which is statistically classified as low accuracy. The diagnostic value of self-diagnosis of influenza was not superior to that of axillary temperature or pulse rate, by AUC evaluation. However, using quantitative rather than qualitative data for diagnosis could be useful owing to the availability of different cut-off points, which allows for a more accurate assessment of signs and symptoms. Our study showed that extremely high or low cut-off points contribute to the diagnosis of influenza (Table 2). Jutel et al. reported a sensitivity of 45.7% (33.0 to 58.3) and specificity of 58.1% (51.0 to 65.3) for detecting seropositive influenza status [10], which would correspond to our data around a 0.5 cut-off point.

The combination of acute or sudden onset fever and cough increased the likelihood ratio to the greatest degree. The LR+ was 3.27 (1.68 to 6.35) (Table 3). Whereas the sensitivity was slightly improved in the subgroup with sudden onset fever, the improvement was insufficient for a definite diagnosis. As Jutel et al. reported [11], the absence of cough reduced the likelihood of influenza, which could be useful for ruling it out. No other clinical signs were found to be useful for ruling in or ruling out influenza in our study.

### Expectations for the advancement of influenza self-diagnosis

Self-medication is strongly related to self-diagnosis. The increasing use of over-the-counter medicines plays an important role in treating mild to moderate influenza, and has been reported to save the US health system US$ 102 billion annually [4]. In 2005, it was reported that the first access for obtaining health information was the Internet for 48.6% of people aged 18 years or older in the U.S.; only 10.9% went first to their physicians [14]. The accuracy of self-diagnosis for various diseases, e.g., urinary tract infection, high blood pressure, vaginal yeast infection, and head lice, has been reported [15–19]. The increasing use of the internet and online self-diagnostic support systems can increase the accuracy of the patient’s diagnosis [20]. In several countries, there is greater availability of various self-diagnostic tests such for pregnancy, HIV, syphilis, hypercholesterolemia, prostate cancer, Alzheimer disease, and malaria [21–25], and self-diagnosis using these tests has become easier. In Japan, there is no available self-diagnostic kit for influenza. Considering the risk of overdiagnosis or misdiagnosis of influenza among patients and physicians, a rational strategy for preventing the spread of influenza could be to integrate self-diagnosis, some clinically useful signs, and test kits that can be used by patients. In 2016, Hannah et al. reported that a diagnosis using the internet was less accurate than that by physician [26]. However, we can predict that developments in artificial intelligence will improve the accuracy of such diagnoses. We expect that more appropriate self-diagnosis and subsequent actions will contribute to not only individual patients’ self-management but also pandemic control, thereby saving healthcare costs and more effectively utilizing health resources worldwide.

### Limitations of the study

Our study had several limitations. Firstly, the study was undertaken in a rural area of Japan. The reliability of medical information was high owing to the somewhat isolated nature of the rural area. However, the result might be different with different conditions, such as 1) region, e.g., urban area, 2) patient background, and 3) influenza epidemic situation. Secondly, we could not use data from prior to December 2013 because the format of the pre-examination checklist was different, and the quality of data could not be verified. Finally, we excluded the data from those pre-examination checklists completed by patients’ parents or caregivers and in which the estimation of self-diagnosis was not recorded in percent figures. The concept of percent is introduced to 10- to 11-year-olds in the fifth grade of elementary school in Japan. It would therefore be necessary to change the method of questioning depending on differences in the educational level and cultural differences of the patients.

## Conclusion

We investigated the accuracy of self-diagnosis of seasonal influenza. Interpreting the accuracy at different cut-off points could be clinically applied. This would be more useful if considered in combination with other clinical signs such as history of fever, severity of feeling ill, and cough.

## Supporting information

S1 Fig Japanese version (Original version) of pre-examination checklist. This checklist was filled out before medical consultation.
(PDF)

S2 Fig English version of pre-examination checklist. Japanese version of pre-examination checklist (S1 Fig) was translated in English.
(PDF)
