# Supplementary material for: Self-diagnosis of seasonal influenza in a rural primary care setting in Japan: A cross sectional observational study
Source: PLoS One. 2018 May 10;13(5):e0197163. doi: 10.1371/journal.pone.0197163 (PMC5944958; doi:10.1371/journal.pone.0197163)
Supplement: S1 Fig — This checklist was filled out before medical consultation. (PDF) [file pone.0197163.s001.pdf]

インフルエンザ疑い患者に対するチェック項目

患者氏名　　　　　　　　　　　　**年齢**　　　**性別**　　M ／ F

体温　　　　　　　℃　　**脈拍**　　　　　　　／min

● **いままでにインフルエンザにかかったことがありますか？**（□にチェックを）

□ はい　　　　　　□ いいえ

● **どのような症状がありますか？**

□ 突然、または急に熱が上昇した　□ ゆっくりと熱が少々してきた　□ 熱はない
□ 咳がある　□ 咳はない
□ 関節痛がある　□ 関節痛はない
□ 筋肉痛がある　□ 筋肉痛はない

● **最初に症状が出現したのはいつですか？**

月　　日　　　午前・午後　　時　　分　　頃

● **いつもの風邪（普段経験する風邪）よりつらいですか？**

□ つらい　　□ 同じくらい　　□ つらくない

● **インフルエンザの可能性はどれくらいだと感じていますか？**

＿＿＿＿＿＿＿＿％（0～100％）
