# Supplementary material for: Self-diagnosis of seasonal influenza in a rural primary care setting in Japan: A cross sectional observational study
Source: PLoS One. 2018 May 10;13(5):e0197163. doi: 10.1371/journal.pone.0197163 (PMC5944958; doi:10.1371/journal.pone.0197163)
Supplement: S2 Fig — Japanese version of pre-examination checklist (S1 Fig) was translated in English. (PDF) [file pone.0197163.s002.pdf]

PRE-EXAMINATION CHECKLIST

NAME ______ AGE ______ SEX M / F

AXILLARY TEMPERATURE ______ ℃ PULSE RATE ______ /min

- **Have you ever had influenza?**

  □ Yes □ No

- **Which of the following symptoms do you have?**

  □ Acute or sudden onset fever □ Gradually onset fever □ No fever
  □ Cough □ No cough
  □ Joint pain □ No joint pain
  □ Muscle pain □ No muscle pain

- **When did your symptoms start?**

  Date ______ (dd) ______ (mm) Time : ______ a.m. / p.m.

- **What is the severity of your symptoms?** (**As compared with a common cold)**

  □ Severe □ Similar □ Mild

- **How do you feel about the possibility of influenza?**

  ______ % (0 – 100%)
